# Supplementary material for: Proteomic Profiling of Plasma- and Gut-Derived Extracellular Vesicles in Obesity
Source: Nutrients. 2024 Mar 4;16(5):736. doi: 10.3390/nu16050736 (PMC10935251; doi:10.3390/nu16050736)
Supplement: Supplementary file 1 [file nutrients-16-00736-s001.zip › nutrients-2838849-supplementary.pdf]

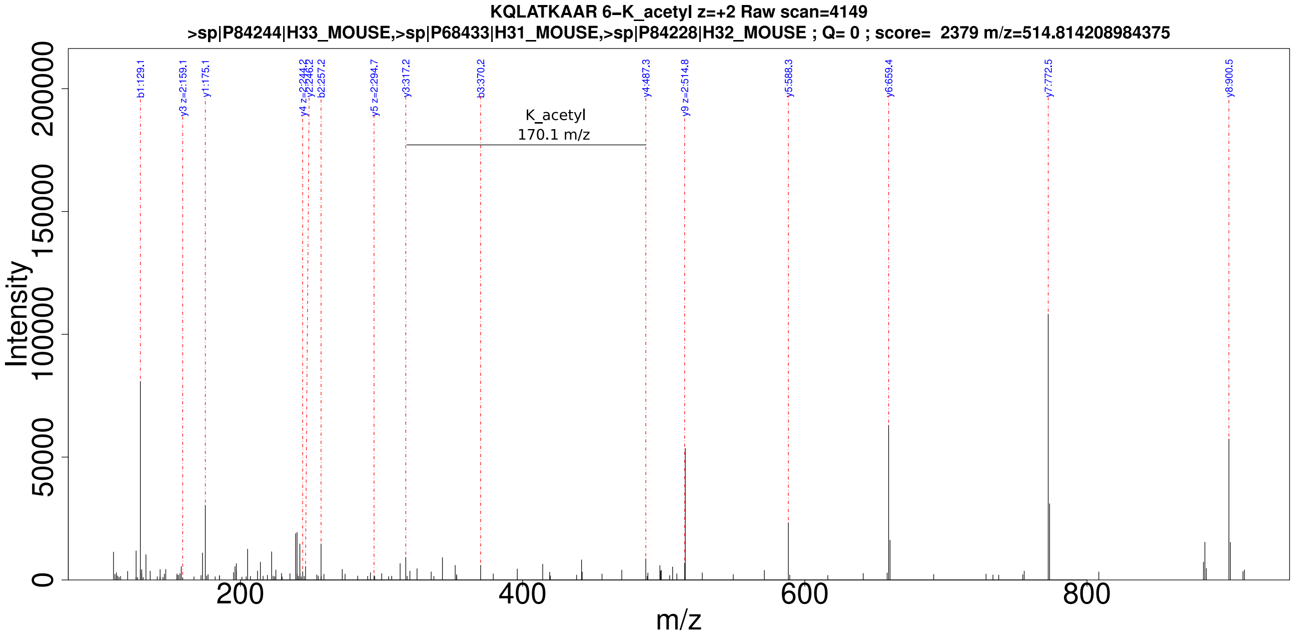

Figure S1: Examples of annotated raw spectrum of identified lysine acetylation site.

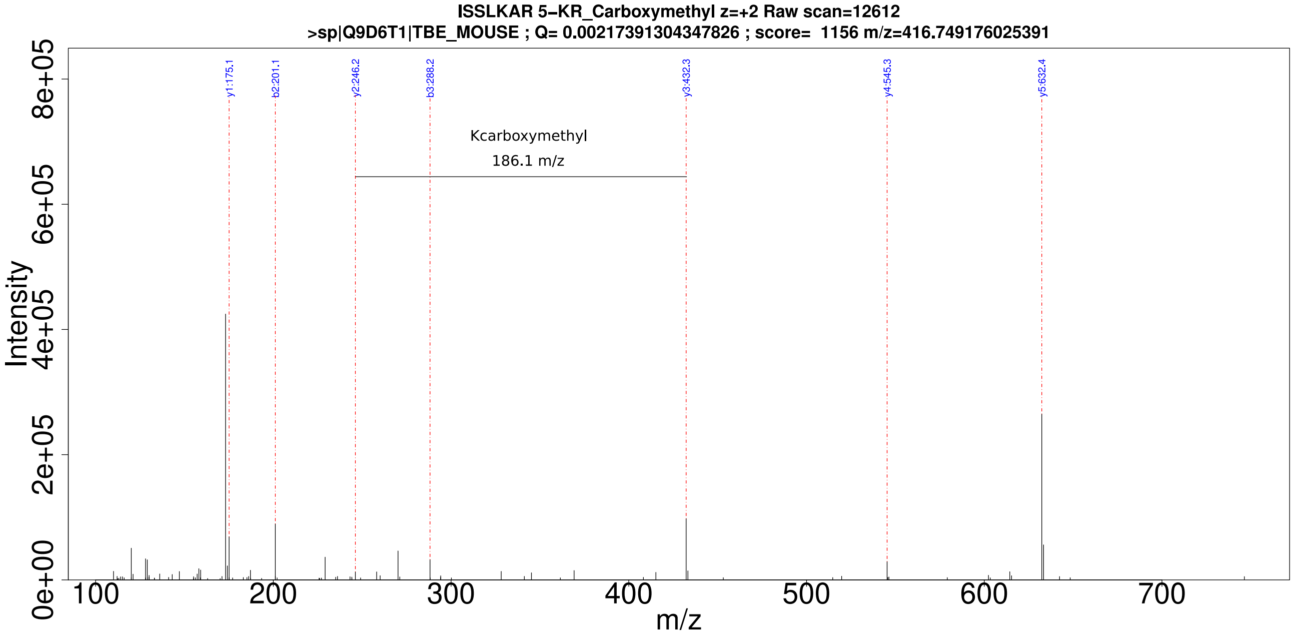

Figure S2: Examples of annotated raw spectrum of identified lysine glycation site.

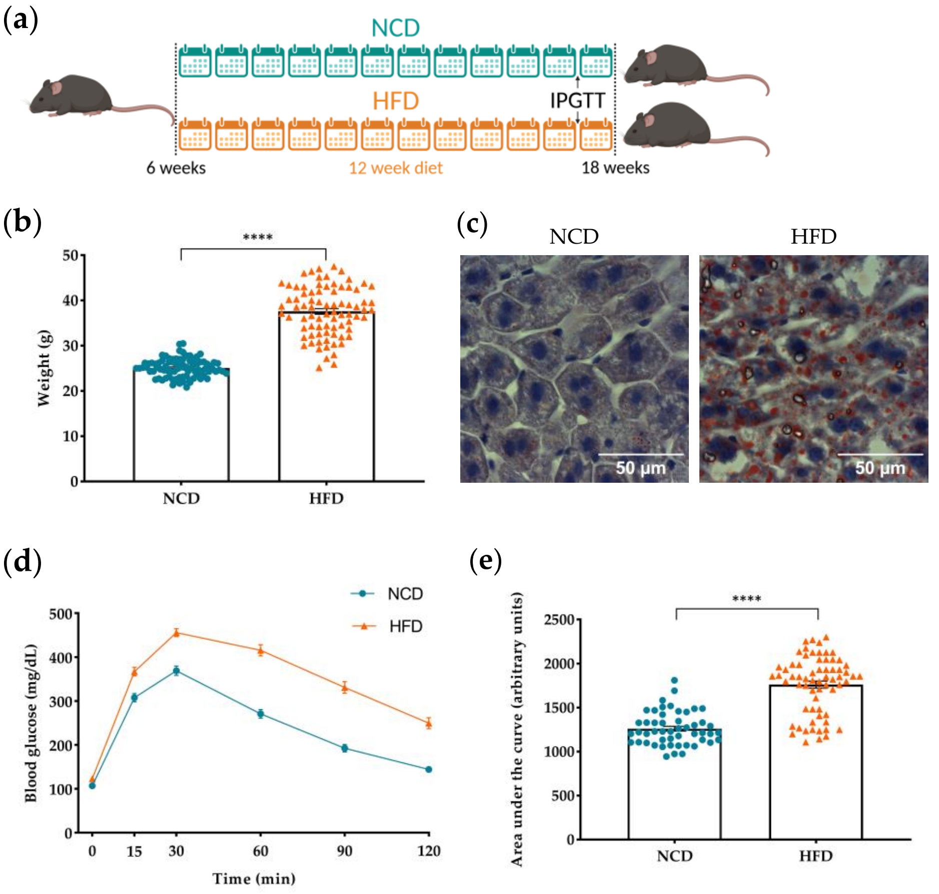

Figure S3: Diet-induced obese mouse model characterization.
(a) Schematic representation of the 12-week diet plan. Obesity was induced in male C57Bl/6J mice by feeding them a high fat diet (HFD), while control mice were fed a normal chow diet (NCD). At the 11th week of diet, mice are subjected to intra-peritoneal glucose tolerance test (ipGTT). **(b)** Statistical analysis of mouse body weight (circles indicate individual animals). **(c)** Hematoxylin and eosin + Oil Red O staining in liver, histological sections of NCD and HFD mice with 63X magnification. **(d)** Representation of ipGTT at different time points after glucose administration (0, 15, 30, 60, 90, 120 minutes). **(e)** Statistical analysis of the area under the curve of the ipGTT graph (circles indicate individual animals; n=60 for NCD and n=70 for HFD).
All statistical analysis were performed using Unpaired t-test with Welch's correction. All data are presented as mean ± standard error of the mean. **** P-value < 0.0001.

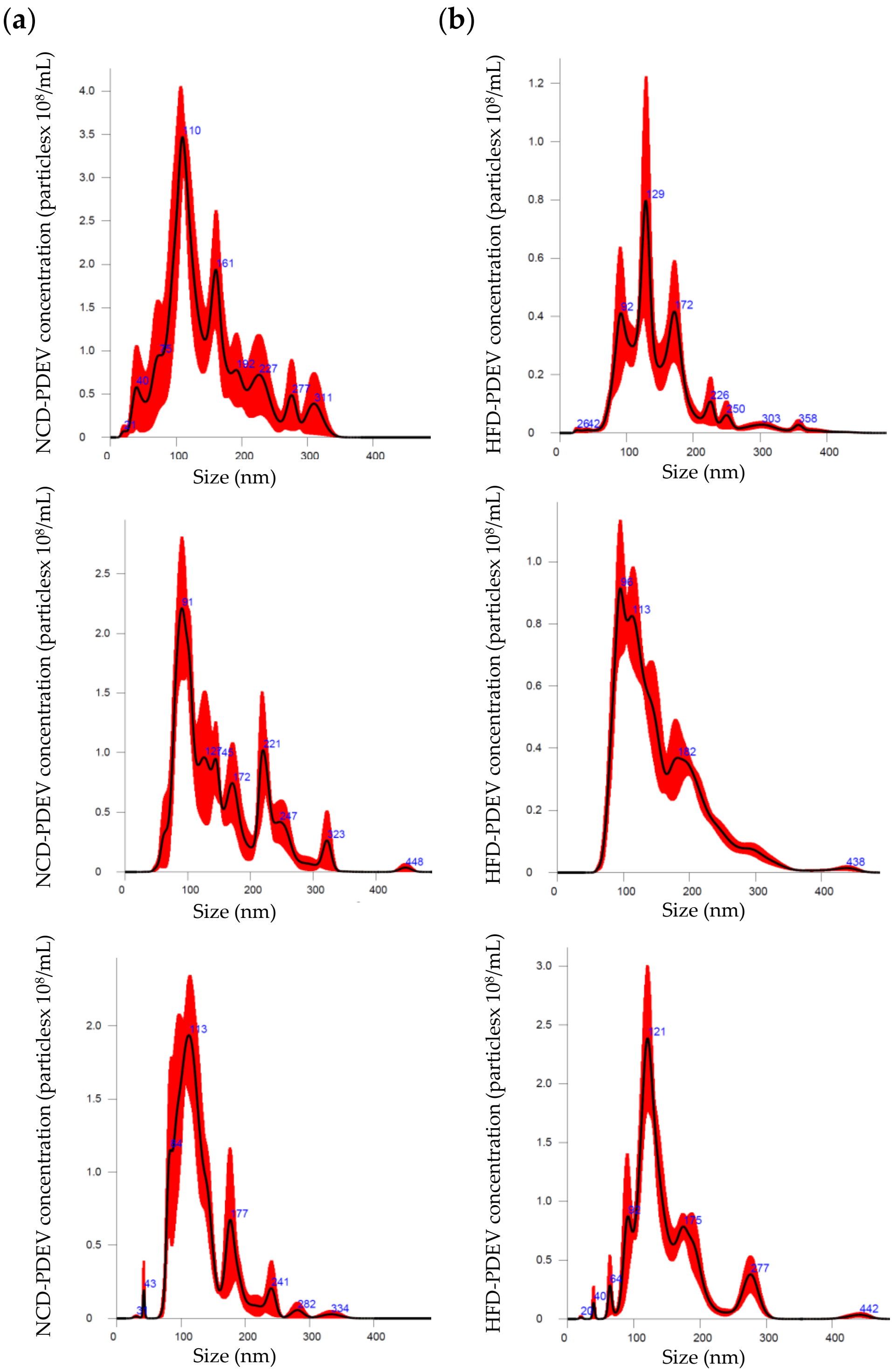

Figure S4: Nanoparticle tracking analysis of Plasma EVs.
Size and concentration distribution of Plasma EVs determined by nanoparticle tracking analysis (NTA). **(a)** NTA graphs of Plasma EVs replicates from NCD mice. **(b)** NTA graphs of Plasma EVs replicates from HFD mice.

Table S1: List of 112 protein exclusively identified in Plasma EVs from NCD mice relative to Figure 2b and Figure 3.

Protein	Gene	False Discovery Rate
14-3-3 protein eta	Ywhah	0
14-3-3 protein gamma	Ywhag	0.00784313725490196
14-3-3 protein theta	Ywhaq	0
4-trimethylaminobutyraldehyde dehydrogenase	Aldh9a1	0
Adenosylhomocysteinase	Ahcy	0
Adenylyl cyclase-associated protein 1	Cap1	0
Adiponectin	Adipoq	0
Afamin	Afm	0
Alcohol dehydrogenase 1	Adh1	0
Alpha-2-HS-glycoprotein	Ahsg	0
Angiopoietin-related protein 6	Angptl6	0
Annexin A7	Anxa7	0
Argininosuccinate synthase	Ass1	0
Basigin	Bsg	0
Bleomycin hydrolase	Blmh	0.00699300699300699
Calmodulin-1	Calm1	0
Calmodulin-2	Calm2	0
Calmodulin-3	Calm3	0
Calmodulin-like protein 3	Calml3	0
Caveolin-1	Cav1	0
Cholinesterase	Bche	0
Coagulation factor XIII B chain	F13b	0
Collectin-11	Colec11	0
Complement factor H	Cfh	0
C-reactive protein	Crp	0
Creatine kinase U-type, mitochondrial	Ckmt1	0
Delta-aminolevulinic acid dehydratase	Alad	0
Endoplasmic reticulum chaperone BiP	Hspa5	0
Estradiol 17 beta-dehydrogenase 5	Akr1c6	0
Estradiol 17-beta-dehydrogenase 11	Hsd17b11	0
Gelsolin	Gsn	0
Glial fibrillary acidic protein	Gfap	0
Glutathione peroxidase 3	Gpx3	0
Glyceraldehyde-3-phosphate dehydrogenase	Gapdh	0
Glyceraldehyde-3-phosphate dehydrogenase, testis-specific	Gapdhs	0
Guanine nucleotide-binding protein G(o) subunit alpha	Gnao1	0
Guanine nucleotide-binding protein subunit alpha-12	Gna12	0
Guanine nucleotide-binding protein subunit alpha-13	Gna13	0
Heat shock 70 kDa protein 1A	Hspa1a	0

Table S1 (continuation): List of 112 protein exclusively identified in Plasma EVs from NCD mice relative to Figure 2b and Figure 3.

Protein	Gene	False Discovery Rate
Heat shock 70 kDa protein 1B	Hspa1b	0
Heat shock 70 kDa protein 1-like	Hspa1l	0
Heat shock cognate 71 kDa protein	Hspa8	0
Heat shock-related 70 kDa protein 2	Hspa2	0
Hemoglobin subunit epsilon-Y2	Hbb-y	0.00706713780918728
Hemoglobin subunit zeta	Hbz	0
Histidine-rich glycoprotein	Hrg	0
Ig gamma-2A chain C region secreted form	-	0
Ig heavy chain V region TEPC 1017	-	0.00701754385964912
Ig heavy chain V region UPC10	-	0
Ig kappa chain V region Mem5 (Fragment)	-	0
Ig kappa chain V-II region MOPC 511	-	0
Ig kappa chain V-II region VKappa167	Gm5153	0
Ig kappa chain V-VI region NQ6-8.3.1	-	0
Inhibitor of carbonic anhydrase	Ica	0
Integrin beta-1	Itgb1	0
Katanin p60 ATPase-containing subunit A-like 2	Katnal2	0.00696864111498258
Keratin, type I cuticular Ha1	Krt31	0
Keratin, type I cuticular Ha2	Krt32	0
Keratin, type I cuticular Ha3-II	Krt33b	0
Keratin, type I cuticular Ha5	Krt35	0
Keratin, type I cuticular Ha6	Krt36	0
Keratin, type I cytoskeletal 24	Krt24	0
Keratin, type I cytoskeletal 25	Krt25	0
Keratin, type I cytoskeletal 27	Krt27	0
Keratin, type I cytoskeletal 28	Krt28	0
Keratin, type I cytoskeletal 40	Krt40	0
Keratin, type II cuticular 87	Krt87	0
Keratin, type II cuticular Hb1	Krt81	0
Keratin, type II cuticular Hb5	Krt85	0
Keratin, type II cuticular Hb6	Krt86	0
Kininogen-1	Kng1	0
Lactadherin	Mfge8	0
Leukocyte surface antigen CD47	Cd47	0
LIM/homeobox protein Lhx2	Lhx2	0
LIM/homeobox protein Lhx9	Lhx9	0
Mannan-binding lectin serine protease 1	Masp1	0
Mannose-binding protein A	Mbl1	0
Microtubule-associated protein 9	Map9	0

Table S1 (continuation): List of 112 protein exclusively identified in Plasma EVs from NCD mice relative to Figure 2b and Figure 3.

Protein	Gene	False Discovery Rate
Myosin light polypeptide 6	Myl6	0.00711743772241993
Myosin-9	Myh9	0
Neurofilament heavy polypeptide	Nefh	0.00704225352112676
Peptidyl-prolyl cis-trans isomerase A	Ppia	0
Peroxiredoxin-2	Prdx2	0
Pituitary tumor-transforming gene 1 protein-interacting protein	Pttg1ip	0
Platelet glycoprotein Ib beta chain	Gp1bb	0.00775193798449612
Properdin	Cfp	0
Proteasome subunit alpha type-4	Psma4	0
Proteasome subunit alpha type-6	Psma6	0
Proteasome subunit beta type-2	Psmb2	0
Proteasome subunit beta type-3	Psmb3	0
Proteasome subunit beta type-4	Psmb4	0
Proteasome subunit beta type-5	Psmb5	0
Proteasome subunit beta type-7	Psmb7	0
Proteasome subunit beta type-8	Psmb8	0
Pyruvate kinase PKM	Pkm	0
Ras-related protein Rab-21	Rab21	0
Ras-related protein Rap-1b	Rap1b	0
Secretoglobin family 2B member 2	Scgb2b2	0
Serine protease inhibitor A3C	Serpina3c	0
Serine protease inhibitor A3F	Serpina3f	0
Serine protease inhibitor A3G	Serpina3g	0
Serine protease inhibitor A3N	Serpina3n	0
Sorcin	Sri	0
Spermatogenesis-associated serine-rich protein 2	Spats2	0
T-complex protein 1 subunit beta	Cct2	0
T-complex protein 1 subunit delta	Cct4	0
T-complex protein 1 subunit gamma	Cct3	0
T-complex protein 1 subunit theta	Cct8	0
TRAF3-interacting protein 1	Traf3ip1	0
Transferrin receptor protein 1	Tfrc	0
Vimentin	Vim	0
von Willebrand factor	Vwf	0

Table S2: List of 4 proteins shared between NCD and HFD plasma EVs, but exclusively acetylated in NCD plasma EVs, relative to the Venn Diagram in Figure 5a.

Proteins	Gene	False Discovery Rate
Actin, cytoplasmic 2	Actg1	0
Immunoglobulin heavy constant mu	Ighm	0.000499875031242189
Major vault protein	Mvp	0
Pregnancy zone protein	Pzp	0

Table S3: List of 6 Proteins shared between NCD and HFD plasma EVs, but exclusively glycated in NCD plasma EVs, relative to the Venn Diagram in Figure 5b.

Proteins	Gene	False Discovery Rate
Ig alpha chain C region	-	0
Ig heavy chain V region 1-62-3	Ighv1-62-3	0.00277777777777778
Ig heavy chain V region 1-72	Ighv1-72	0.00277777777777778
Immunoglobulin kappa constant	Igkc	0.00252684775742262
Pregnancy zone protein	Pzp	0
Serum albumin	Alb	0.00715563506261181

Table S4: List of 41 proteins shared between NCD and HFD gut EVs, but exclusively acetylated in NCD gut EVs, relative to the Venn Diagram in Figure 5c.

Proteins	Gene	False Discovery Rate
14-3-3 protein zeta/delta	Ywhaz	0.00929639081297849
Actin-related protein 2/3 complex subunit 1A	Arpc1a	0
Adenylosuccinate synthetase isozyme 2	Adss	0.00227473777328447
Adenylyl cyclase-associated protein 1	Cap1	0
Alanine--tRNA ligase, cytoplasmic	Aars	0.00592665761205087
Arginine--tRNA ligase, cytoplasmic	Rars	0.0033945378799568
Bifunctional glutamate/proline--tRNA ligase	Eprs	0
Calcium/calmodulin-dependent protein kinase type II subunit delta	Camk2d	0.0066119750214277
Calponin-1	Cnn1	0.00268296318378298
cGMP-dependent protein kinase 1	Prkg1	0.00400266844563042
Chloride intracellular channel protein 4	Clic4	0.00241879751209399
Cofilin-2	Cfl2	0
Dynactin subunit 3	Dctn3	0.000326850792613172
Eukaryotic initiation factor 4A-III	Eif4a3	0
Eukaryotic translation initiation factor 3 subunit E	Eif3e	0
Fatty acid-binding protein, adipocyte	Fabp4	0
F-box-like/WD repeat-containing protein TBL1XR1	Tbl1xr1	0
Ferritin light chain 1	Ftl1	0.00239005736137667
Ferritin light chain 2	Ftl2	0.00239005736137667
Glutathione synthetase	Gss	0
Glycogen phosphorylase, liver form	Pygl	0.00788314398330628
Ketosamine-3-kinase	Fn3krp	0.00182815356489945
LIM and senescent cell antigen-like-containing domain protein 2	Lims2	0.00524590163934426
MOB kinase activator 1B	Mob1b	0.00713545862584877
N-acetyl-D-glucosamine kinase	Nagk	0
Nucleoside diphosphate kinase B	Nme2	0.00681609647398086
Phosphoglycerate kinase 1	Pgk1	0.00225790265930758
Protein SGT1 homolog	Sugt1	0
Purine nucleoside phosphorylase	Pnp	0.00247875354107649
Ribonuclease inhibitor	Rnh1	0
RNA polymerase-associated protein RTF1 homolog	Rtf1	0.00136495478587272
Serine/threonine-protein phosphatase 2B catalytic subunit beta isoform	Ppp3cb	0.00884857869704679
Serine/threonine-protein phosphatase CPPED1	Cpped1	0
Signal transducer and activator of transcription 1	Stat1	0
START domain-containing protein 10	Stard10	0.0022312090363966
Sulfotransferase family cytosolic 1B member 1	Sult1b1	0.00370313223268014
Thyroid hormone-inducible hepatic protein	Thrsp	0
Transitional endoplasmic reticulum ATPase	Vcp	0
Tripeptidyl-peptidase 2	Tpp2	0.0069609818648104
Ubiquitin-conjugating enzyme E2 variant 1	Ube2v1	0
UDP-N-acetylhexosamine pyrophosphorylase	Uap1	0.00226986128625473

Table S5: List of 29 proteins shared between NCD and HFD gut EVs, but exclusively acetylated in HFD gut EVs, relative to the Venn Diagram in Figure 5c and Figure 6.

Proteins	Gene	False Discovery Rate
Acyl-coenzyme A thioesterase 4	Acot4	0.00382667416994935
Alpha-enolase	Eno1	0
Alpha-mannosidase 2C1	Man2c1	0.000421496311907271
COP9 signalosome complex subunit 3	Cops3	0
DNA-directed RNA polymerases I, II, and III subunit RPABC3	Polr2h	0
Endoribonuclease LACTB2	Lactb2	0.00104675505931612
F-box only protein 22	Fbxo22	0.00901135083614938
Galectin-2	Lgals2	0.00903274369589763
Gelsolin	Gsn	0.00206947524020695
Glutathione S-transferase A1	Gsta1	0.000643500643500644
Glutathione S-transferase A2	Gsta2	0.000643500643500644
GMP synthase [glutamine-hydrolyzing]	Gmps	0.00158982511923688
Histone H2B type 1-C/E/G	Hist1h2bc	0
Histone H2B type 1-F/J/L	Hist1h2bf	0
Histone H2B type 1-K	Hist1h2bk	0
Histone H2B type 1-M	Hist1h2bm	0
Histone H2B type 1-P	Hist1h2bp	0
Histone H2B type 2-B	Hist2h2bb	0.000985059924478739
Histone H2B type 2-E	Hist2h2be	0
Histone H2B type 3-B	Hist3h2bb	0
Histone H3.3C	H3f3c	0
Histone H4	Hist1h4a	0
Nuclear protein localization protein 4 homolog	Nploc4	0.00573065902578797
Peptidyl-prolyl cis-trans isomerase D	Ppid	0
Phosphoribosyl pyrophosphate synthase-associated protein 1	Prpsap1	0.00719144800777454
Proteasome assembly chaperone 3	Psmg3	0.0093143596377749
Staphylococcal nuclease domain-containing protein 1	Snd1	0
Ubiquitin-fold modifier-conjugating enzyme 1	Ufc1	0.00932528798683489
WD repeat-containing protein 61	Wdr61	0.00367225154923112

Table S6: List of 23 proteins shared between NCD and HFD gut EVs, but exclusively glycated in NCD gut EVs, relative to the Venn Diagram in Figure 5d and Figure 6.

Proteins	Gene	False Discovery Rate
Actin, alpha cardiac muscle 1	Actc1	0.00101643232254786
Actin, aortic smooth muscle	Acta2	0.00101643232254786
Actin, gamma-enteric smooth muscle	Actg2	0.00101643232254786
Alcohol dehydrogenase 1	Adh1	0.00989779451317913
AMP deaminase 2	Ampd2	0.00973957230573788
Complement C3	C3	0.00164122763827343
Ferritin heavy chain	Fth1	0.00346658000216661
Fructose-bisphosphate aldolase B	Aldob	0.00392097722817071
Histone H2B type 1-B	Hist1h2bb	0.00688182249644044
Histone H2B type 1-C/E/G	Hist1h2bc	0.00688182249644044
Histone H2B type 1-F/J/L	Hist1h2bf	0.00688182249644044
Histone H2B type 1-H	Hist1h2bh	0.00688182249644044
Histone H2B type 1-K	Hist1h2bk	0.00688182249644044
Histone H2B type 1-M	Hist1h2bm	0.00688182249644044
Histone H2B type 1-P	Hist1h2bp	0.00688182249644044
Histone H2B type 2-B	Hist2h2bb	0.00688182249644044
Host cell factor 1	Hcfc1	0
Selenium-binding protein 2	Selenbp2	0
Serine hydroxymethyltransferase, mitochondrial	Shmt2	0.00891304347826087
Serum albumin	Alb	0.00221647580347248
Thrombospondin-1	Thbs1	0.00710276091190285
Transketolase	Tkt	0.00529567519858782
Valine--tRNA ligase	Vars	0.00247480996994874

Table S7: List of 22 proteins shared between NCD and HFD gut EVs, but exclusively glycated in HFD gut EVs, relative to the Venn Diagram in Figure 5d and Figure 6.

Proteins	Gene	False Discovery Rate
14-3-3 protein zeta/delta	Ywhaz	0.00903725562523054
60S ribosomal protein L5	Rpl5	0.00813522552652987
Actin, cytoplasmic 1	Actb	0.00354649229746204
Actin, cytoplasmic 2	Actg1	0.00354649229746204
Aldehyde dehydrogenase family 1 member A3	Aldh1a3	0.00462737457379445
Alpha-2-macroglobulin-P	A2m	0.00442323361657549
Alpha-actinin-1	Actn1	0.0070168404170008
Apoptosis-inducing factor 1, mitochondrial	Aifm1	0.00646818225054694
Beta-enolase	Eno3	0
Fermitin family homolog 2	Fermt2	0.00973041952464508
Keratin, type II cytoskeletal 8	Krt8	0.00550672455787356
Malate dehydrogenase, cytoplasmic	Mdh1	0.000381825124093165
Myosin-10	Myh10	0.00222129668193808
Pyruvate kinase PKM	Pkm	0.00444912773679897
Regulator of G-protein signaling 18	Rgs18	0.00788484459521408
Serine/threonine-protein kinase VRK1	Vrk1	0.00582120582120582
Tetratricopeptide repeat protein 38	Ttc38	0
Tubulin alpha-1A chain	Tuba1a	0.00168705187684521
Tubulin alpha-1B chain	Tuba1b	0.00168705187684521
Tubulin alpha-3 chain	Tuba3a	0.00168705187684521
Tubulin alpha-4A chain	Tuba4a	0.00168705187684521
Tyrosine--tRNA ligase, cytoplasmic	Yars	0.00807511737089202

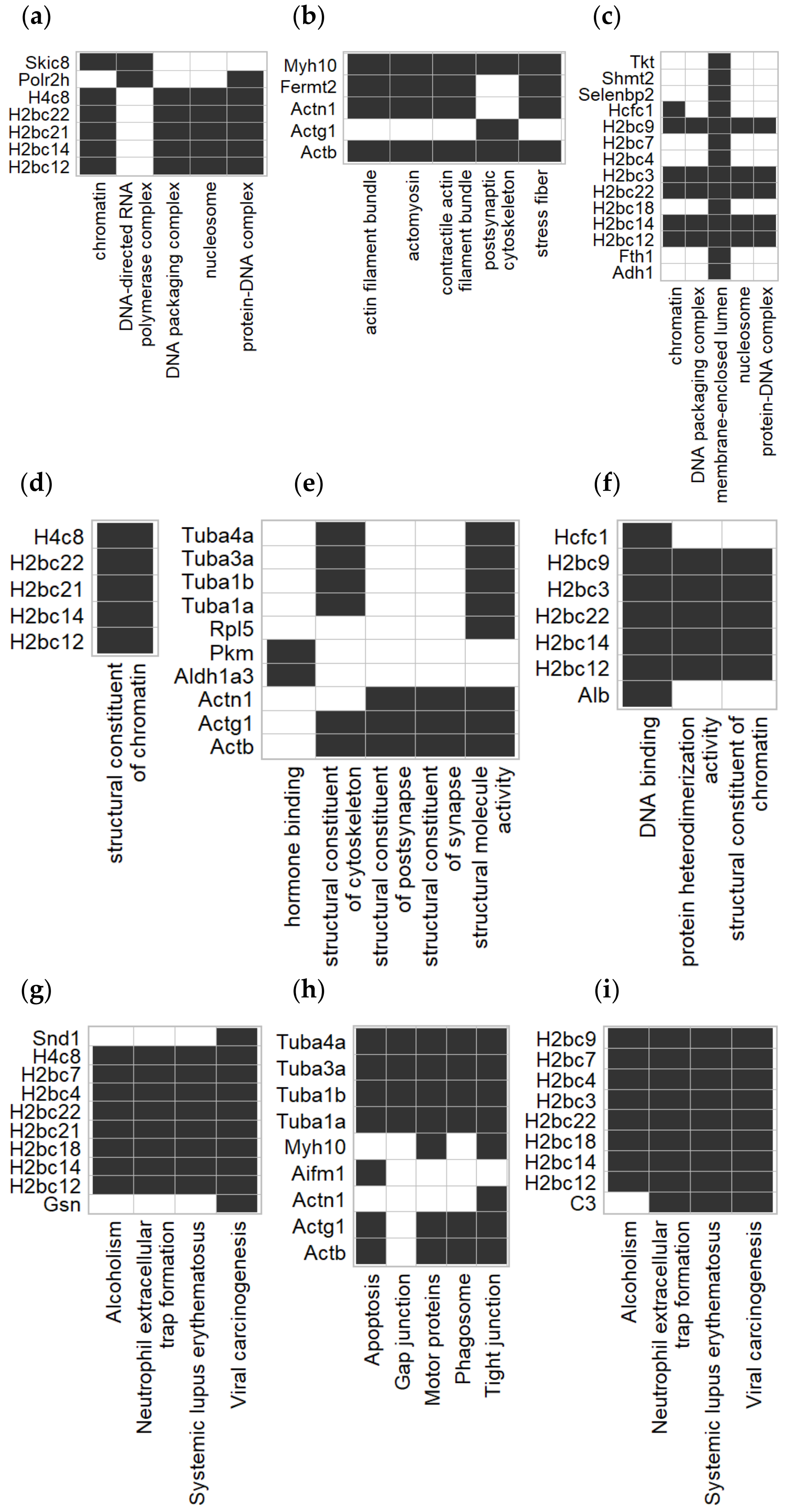

Figure S5: Enriched terms and their related identified genes, relative to Figure 6.
(a) GO-Cellular Components enrichment analysis of acetylated proteins in HFD gut EVs. **(b)** GO-Cellular Components enrichment analysis of glycated proteins in HFD gut EVs. **(c)** GO-Cellular Components enrichment analysis of glycated proteins in NCD gut EVs. **(d)** GO-Molecular Functions enrichment analysis of acetylated proteins in HFD gut EVs. **(e)** GO-Molecular Functions enrichment analysis of glycated proteins in HFD gut EVs. **(f)** GO-Molecular Functions enrichment analysis of glycated proteins in NCD gut EVs. **(g)** KEGG Pathways enrichment analysis of acetylated proteins in HFD gut EVs. **(h)** KEGG Pathways enrichment analysis of glycated proteins in HFD gut EVs. **(i)** KEGG Pathways enrichment analysis of glycated proteins in NCD gut EVs.
Black rectangles indicate the association between the identified gene and the enriched term.
